# The Role of Teacher–Student Relationships in Predicting Teachers’ Occupational Wellbeing, Emotional Exhaustion, and Enthusiasm

**DOI:** 10.3389/fpsyg.2022.896813

**Published:** 2022-05-19

**Authors:** Liying Cui

**Affiliations:** School of Marxism, Southeast University, Nanjing, China

**Keywords:** emotional exhaustion, enthusiasm, teacher–student relationships, teachers’ occupational wellbeing, educator burnout

## Abstract

Wellbeing is regarded as a core dimension of an individual’s prosperity in the field of positive psychology. Underlying this multifaceted framework are emotive, mental, and societal forms of wellbeing, which can be based on constructive connections in the workplace. Career wellbeing among educators is linked to optimal mental functioning and their positive career experience is characterized in terms of the existence of constructive dimensions like enthusiasm at the workplace. Also, emotional exhaustion or fatigue is another central element in the research on educator burnout and it has an important effect on educators’ expert lives, alternatively, the excellence of teacher–student relationships is significant in the process of language learning. Due to the significant effect of the above-mentioned construct, this review tries to focus on the prominence of teacher–student relationships in this domain. The results from the review of the literature specified that high-value teacher–student relationships keep educators away from being emotionally exhausted since it can enhance the quantity of enthusiasm and lessen the amount of exhaustion. In a nutshell, this review of literature has suggestions for academics and experts in search of increasing teachers’ occupational wellbeing.

## Introduction

All professions have dimensions that increase motivation and participation in the workplace or threaten wellbeing. In many professions including education which is a very demanding and stressful career, researchers have studied negative aspects of employment, work-related stress, and exhaustion (Hakanen et al., 2006; Kokkinos, 2007). Everyone knows that teaching is a difficult and occasionally boring occupation. High rates of dropout and untimely retiring of educators in recent years have raised some public concern and led to research into educator burnout as a probable reason beyond educator attrition (Chang, 2009). When educators worldwide are facing new difficulties and unexpected job fluctuations because of various problems and adversities in the education process, education organization express their concerns regarding educators’ wellbeing (Fathi et al., 2020). By choosing an interpretation system that focuses on these concerns, scholars can emphasize factors that vigorously aid in maintaining the cycle of prosperity, as opposed to deconstructive environmental characteristics that must be evaded as much as possible (Xie and Derakhshan, 2021).

A significant amount of literature shows that the professional wellbeing of educators is important for both themselves and their learners (Madigan and Kim, 2021). In previous decades, there has been a renewed awareness of the professional wellbeing of educators in the field of educational studies (Cumming, 2017). In other words, both theoretically and practically, educators’ occupational wellbeing including emotional and work fatigue is a vital issue in this field. This has to do with the attrition and intellectual and physical health of educators, along with learner motivation and achievement (Klusmann et al., 2016). Indeed, negative dimensions of professional wellbeing, like experiencing stress at work or feeling emotional fatigue, put pressure on educators; however, the positive dimensions of occupational wellbeing can protect them against harmful factors of wellbeing and it may protect the fundamentals that are detrimental and destructive for their wellbeing (Bermejo-Toro et al., 2016). Many scholars suggest that emotional fatigue theoretically lies on the other side of participation in the shared wellbeing continuum of the workplace (Demerouti et al., 2010; Klusmann et al., 2016). Affective exhaustion is described as fatigue and emotional exhaustion, while engagement alludes to working energy, passion, and commitment (Schaufeli and Bakker, 2004).

Moreover, enthusiasm is one of the other remarkable elements in career involvement, or it could be simply a shape of career involvement (Hakanen et al., 2006). Indeed, enthusiasm is described as a constructive emotional inspirational satisfaction incorporating the component of interest (Kunter and Holzberger, 2014). Similarly, enthusiasm is a private encounter of feeling lively and stimulated at work and the display of enthusiasm has been characterized as the attached incidence of constructive emotional encounters and pleasure (Keller et al., 2016). In the same vein, it is believed that educator enthusiasm has a constructive impact on learners’ education, presentation, inspiration, as well as the standard of instruction (Frenzel et al., 2009; Keller et al., 2016). Alternatively, constructive connections with students, guardians, co-workers, and faculty administration seem to promote a constructive mindset in the direction of the career, enhance educators’ degree of inspiration and involvement in their work, and enhance their tendency to work on themselves in building their expert skills (Buonomo et al., 2017). Indeed, for beginner educators’ career wellbeing; social interactions with learners are especially relevant (Schmidt et al., 2017).

Lots of evidence revealed that learner delinquency and challenges in the classroom, such as learner motivation, coping with heterogeneity, or building rapport with learners, are major stressors in these people (Aldrup et al., 2018; Xie and Derakhshan, 2021). Therefore, it can be said that establishing positive relationships between educators and learners is the main purpose of educators. Based on the stress-coping transactional model, failure in achieving this purpose straightly pertains to higher degrees of stress and lower wellbeing (Chaplain, 2008; Aldrup et al., 2018). The Educator–learner relationship is a mediator, that is, learner misbehavior conveys a feeling of rejection to the educators and prevents them from establishing emotional relationships with learners (Spilt et al., 2011). Failure to achieve this goal can be detrimental to educators’ wellbeing because building positive educator–learner relationships is a primary goal for educators (Newberry and Davis, 2008; Butler, 2012; Nurmi and Kiuru, 2015).

Conversely, positive interactions and relationships between educators and learners can serve as a source to keep wellbeing and avoid emotional fatigue (Spilt et al., 2011). Aldrup et al. (2018) indicated that novice educators specifically can have encouraging connections with learners, and positive connections were found to reduce their emotional fatigue. Educators, however, must address numerous difficulties and demands in the classroom that can endanger their professional wellbeing and, consequently, leave a negative effect on their capability to generate a supportive learning setting and have supported student interaction (Friedman-Krauss et al., 2014; Buettner et al., 2016).

Based on attachment and self-determination theories, positive educator–learner relationships are keys to the development of learners. This is because educators who guide and support learner behavior in academic and emotional terms build a social context in which learners feel secure, interconnected, independent, competent, and motivated to learn, capable of personal growth (Aldrup et al., 2018). Many empirical studies are consistent with this assumption and show that educator proximity and educator influence are related to individual classroom outcomes, such as learner accomplishment, self-esteem, interest, or effort (Wentzel et al., 2010; Scherer et al., 2016). Nevertheless, these investigations have hardly ever inspected how educators influence their occupational wellbeing.

Educators who felt associated with their learners have been more prone to record pleasure and less prone to report stress and rage. In conformity with the hypothesis of attachment, constructive relational connections mirror protection, and so, have a significant function for learners and work as a precursory for educators’ wellbeing (Kyriacou, 2001). Nonetheless, scholars up until now have emphasized the constructive effect educator–learner connections have on learners and have greatly ignored its possible influences on educators (Roorda et al., 2011). Certainly, feeling linked to learners has proven to be deconstructively connected to educators’ degree of emotive fatigue (Klassen et al., 2012); for this reason, it appears that educators who experience proximity with their learners will display lower degrees of emotive fatigue than educators who do not have a sense of proximity with their learners. For educators, these relations are associated with job gratification, teacher wellbeing, and low degrees of trauma and tension (Gu and Day, 2007; Veldman et al., 2013).

The literature explains the high standard connections as cordial and welcoming. In these connections, educators develop an organized setting with evident anticipations while, at the same time, passing on a message of rapport and reciprocated regard (Wubbels et al., 2015). Alternatively, relationships with low quality indicate high levels of educators–learners conflict and disagreement, which educators describe as conflicting, or distant. Due to the effect of these relationships on learner–educator results, understanding the basics of these relationships is significant (Spilt et al., 2011). Indeed, because of the impact that these relationships have on student and teacher upshots, it is central to investigate them in detail. So, it is claimed that if good teacher–student relations work as a defensive issue toward their wellbeing through their impact on educators’ eagerness and exhaustion, this paper makes an effort to consider this research lacuna by inspecting the role of teachers’ enthusiasm and emotional exhaustion as subcomponents of educators’ occupational wellbeing can foster the connection between teacher–student relationships.

## Review of the Literature

### Teacher–Student Relationship

A constructive educator–learner connection is usually described by regard, compassion, faith, and low degrees of relational dispute (Roorda et al., 2011). Grounded on attachment and self-determination theories, such qualities are deemed essential for the growth of learners as they offer a sense of security and belonging. Vast empirical evidence indicates that learners who receive an educator’s appreciation and support achieve more positive emotional, behavioral, and cognitive results (McGrath and van Bergen, 2015; Vandenbroucke et al., 2018). Educator–learner relationships generally feature reciprocal respect, trust, warmth, and low levels of contradiction (Aldrup et al., 2018). This relationship has a central role in the development of learners as it conveys to learners a sense of safety and attachment to their educator and provides learners with the sense of educator’s appreciation and support and ultimately, they are motivated to achieve positive cognitive, emotional, and behavioral learning results (Kunter et al., 2013; Aldrup et al., 2018). In case of such a relationship, educators become passionate and motivated to teach learners and advise them, where ultimately learning goals will be optimally achieved. Also, such relationships will lower the emotional exhaustion of the educator, because when the educator feels passionate and enthusiastic about working, the stress is automatically lowered when in a psychological state (Aldrup et al., 2018).

Furthermore, a theoretical model of educator wellbeing is built that highlights the significance of the educator–learner relationship, proposed as a mediator between learner misconduct and educator wellbeing (Spilt et al., 2011). To be exact, learner misconduct impairs the relationship between educator–learner because it induces destructive connections and can be understood as the absence of educator appreciation (Spilt et al., 2011). Also, recently, the research found that educators who had higher relationships with learners were more passionate about their work, however, no association was found with their emotional exhaustion (Aldrup et al., 2017).

### Teachers’ Occupational Wellbeing

The notions of wellbeing and career wellbeing have been utilized highly differently in the literature. In the present research, the elements that influence career wellbeing and its advancement were categorized into four areas: (1) employees and labor, (2) labor circumstance, (3) expert ability, and (4) the labor society. The first area describes employees and their jobs and discusses their psychological and physical workload, personal assets, and influencing elements. Next, the labor circumstances area discusses the physical functioning setting and occupational safety matters. The expert ability area discusses career ability and choices for extra learning or coaching. The area of work community is regarded to incorporate dimensions like career and organizational oversight, leadership, societal help, and employee association (Saaranen et al., 2007).

The occupational or professional wellbeing of educators is a complicated phenomenon approachable from many different perspectives (Cumming, 2017). Its definition is optimal psychological functioning and work experience in which positive aspects like job satisfaction and passion for work are noted and negative experiences are absent like tension and emotional fatigue (Ryan and Deci, 2001). In one aspect, by concentrating on experiences that reduce wellbeing, like work stress, emotional fatigue, or burnout, education has come to recognize the professional wellbeing of educators (Foley and Murphy, 2015). On the other aspect, investigating experiences that can improve wellbeing, like engagement with work, coping tactics, or work recovery on can gain valuable knowledge (Granziera and Perera, 2019; Virtanen et al., 2020; Greenier et al., 2021). Prolonged work-related stress leads to burnout and consists of three symptoms: emotional fatigue, cynicism, and a subjective sense of inefficiency. Emotional exhaustion explains the stressful aspect of burnout, which encompasses tension and exhaustion of emotional resources, usually used as an indicator of educators’ (diminished) professional wellbeing (Maslach et al., 2001).

Emotional exhaustion defines the stress element of burnout and contains an emotional state of anxiety and the exhaustion of one’s emotive sources and it is frequently employed as a sign of educators’ working wellbeing (Dicke et al., 2015; Voss et al., 2017) Cynicism refers to developing an attitude of getting distant from job and indifferent. Emotional fatigue negatively impacts learner achievement as it diminishes learner participation, satisfaction with school, and performance. On the contrary, labor enthusiasm refers to the joy, enjoyment, and delightfulness in their profession as educators (Shen et al., 2015; Klusmann et al., 2016). Contrastingly, work enthusiasm alludes to educators’ pleasure, enthusiasm, and happiness in their work as educators (Kunter et al., 2013). Educators who are passionate about their job can motivate learners and improve learning results (Keller et al., 2014).

### Emotional Exhaustion

Burnout is a long-term reaction to chronic affective and interpersonal stressing factors at work (Maslach, 2003). Undoubtedly, there is a consensus on three main elements of burnout experience, namely, emotional exhaustion, depersonalization, and lower personal achievement. The most important of the three elements of burnout is emotional exhaustion which occurs first during the progress of burnout, and sequentially leads to higher levels of depersonalization and lowered sense of individual fulfillment (Maslach et al., 2001). A sense of emotional exhaustion and lowered emotional resources is the definition provided for emotional exhaustion. Depersonalization is described as the sense of cognitive distance, disregard, or cynicism regarding the person’s work service clients. A decline in personal achievement includes a sense of inefficiency in successfully finishing work requirements and a lack of personal achievement sense (Arens and Morin, 2016). The depersonalization element shows the burnout interpersonal context dimension, indicating a negative or overly segregated reaction to multiple aspects of work. Cynicism refers to efforts made to establish a distance between yourself and the service clients through active overlooking of the traits making them distinctive and attractive (Maslach et al., 2001). Educator burnout is a deconstructive indicator of improving the standard of learner–educator relationships (Hoglund et al., 2015). Correspondingly, Hamre et al. (2008) described a constructive link between educator sadness and learner–educator disputes. Yoon (2002) demonstrated a link between educators’ degrees of anxiety and the rate of deconstructive learner–educator relationships. Ultimately, Jennings and Greenberg (2009) emphasized the contrast of educators’ burnout and mentioned the function of educators’ wellbeing in the projection of healthy learner–educator relationships that are, in turn, known to foresee learners’ results, such as success and inspiration.

### Teacher Enthusiasm

Passion as a positive emotional state is associated with studies of positive psychology (PP) that highlight positive phenomena and emotions. When categorizing the strengths of characters in PP, passion is also one of the forms of the vitality of the strengths of characters (Seligman and Csikszentmihalyi, 2014). In the arrangement of personality assets in PP, enthusiasm is regarded as one type of character strength vigor and the combination of positive emotional experiences and behavioral expressions of such teaching experiences refers to educator passion (Keller et al., 2016). Educator passion is therefore a specific teaching practice to enhance learner motivation and participation. Thus, it has been pointed out in a large body of studies that passionate teaching behaviors positively pertain to learners’ intrinsic motivation, interest, active learning and participation, and enjoyment levels (Frenzel et al., 2009; Keller et al., 2014). Considering a dimension of career involvement, enthusiasm involves emotive and intellectual aspects (Keller et al., 2016).

Work involvement may be characterized as an extensive constructive condition that does not emphasize only one matter, occurrence, or manner (Schaufeli and Bakker, 2004), like school subject or instructing circumstance. Educators who are enthusiastic regarding their class and instruction offer greater help to their learners, thereby leaving a constructive impact on learners’ inspiration (Kunter et al., 2013). Particularly, instructors who are enthusiastic in the classroom might also strengthen learners’ inspiration by offering mastery-directed exercises. Mastery of objective direction in the classroom is described as an emphasis on learners’ education and comprehension and strengthens their inspiration (Meece et al., 2006).

## Conclusion

Maintaining interaction between educators and learners is crucial. This is because this appears to be manifested in the professional wellbeing of educators. Carrying out professional development activities on educator–learner interaction is a possible approach because lowering the stress of educators is significant for engaging them in work, nurturing passion, and encouraging self-reflection and awareness of stress (Sandilos et al., 2018) that also lead to the promotion of teachers’ occupational wellbeing. This review indicated that Positive educator–learner relationships are associated with enhanced work passion and lower emotional fatigue, which is especially important since theory and research in educator wellbeing disregarded the reality that positive educator–learner relationships not only pertain to more positive learner growth (Roorda et al., 2011) but it may be also important for educators’ professional wellbeing (Spilt et al., 2011).

According to the literature review, educator enthusiasm could be very critical for both educators and learners. Moreover, the work wellbeing of an educator is associated with their enthusiasm, and enthusiastic educators are prone to be more fulfilled in their life and at their workplace. Educators with a great degree of enthusiasm do not experience emotive fatigue; therefore, a sense of enthusiasm is constructively connected to health, educators’ joy, and standard of instruction (Kunter et al., 2011). Furthermore, previous investigations authorize that constructive teacher–student relationships will upturn teachers’ work interests and decrease their emotional exhaustion (Spilt et al., 2011; Aldrup et al., 2018). Constructive connections with students, guardians, co-workers, and faculty administration seem to promote a constructive mindset in the direction of the career, enhance educators’ degree of inspiration and involvement in their work, and enhance their tendency to work on themselves in building their expert skills. Positive teacher–student relationships will also reduce teacher emotional exhaustion because as soon as the educator feels enthusiastic and keen on working, it spontaneously decreases and even lessens anxiety and tension in the emotional context. Passionate educators always have a sense of achievement, satisfaction, and happiness because they love their job.

Appropriate teacher–student relationships have been labeled as helpful in avoiding teacher burnout and influential for their work engagement (Doménech-Betoret et al., 2015). Due to the remarkable role of teacher–student relationships in the language classroom, this is worth paying attention to in educator training programs and professional development programs. Investigations have revealed the positive impact of programs focused on educator–learner relationships (Roorda et al., 2011). Thinking about their positive and troublesome relationships is the first stage in developing knowledge of educators on the subject which raises awareness of educators’ unique assumptions and biases in the advent of educator–learner relationships.

This review highpoint the advantages that educators obtain from starting good relationships with their learners. Additionally, educators who are in constructive interactions with their learners are inclined to be interested in their job (Klassen et al., 2012) and it has been stated that educators who participate in noble interactions with their learners report greater degrees of wellbeing and less anxiety (Jennings and Greenberg, 2009). Constructive educator–learner connections are both advantageous to learners, as proved by past studies (Roorda et al., 2011), and are connected to educators being more inspired, which is associated with educators’ encountering lower degrees of emotive fatigue. Furthermore, it appears that concentrating on educators’ and learners’ affinity inside the class would be a favorable approach to lessen educators’ emotive fatigue. The deconstructive connection between educators’ emotive fatigue and learners’ recognition of educator support is possible because educators with great degrees of emotive fatigue do not have enough and sufficient assets to develop constructive and helpful learner–educator relationships (Yoon, 2002; Jennings and Greenberg, 2009).

## Suggestions for Future Studies

One of the main outcomes derived from the current review is that comprehending the elements that keep instructors safe from encountering emotive fatigue could help in lowering the number of educators who quit their careers because of feeling passionately over-extended. In this review study, it is shown that educator–learner connections have a significant function in indirectly lowering educators’ degree of emotive fatigue by elevating their wellbeing. Studies demonstrate that emotively fatigued educators are prone to move away from constructive learner–educator relationships and offer insufficient help to their learners (Chang, 2009).

As some previous studies have shown, a negative school atmosphere can threaten educators’ professional wellbeing (Collie et al., 2012; McLean et al., 2017), educator wellbeing can be maintained through proper relationships and trust between peers and supervisors, adequate resources, and helping to improve learner behavior, and offering opportunities to participate in school decision-making and activities of professional development (Collie et al., 2012). In line with the review of literature, it was revealed that teacher–student relations are a constructive basis for educators in that they incidentally lessen educators’ level of emotional exhaustion.

Educator trainers need to propose interferences to enhance educators’ capability of developing constructive educator–learner connections as it could be advantageous to educators’ career wellbeing. Educator training strategies must try to increase educators’ recognition of the role of particular emotive control techniques, in specific authentically articulated feelings, which portray constructive relationships to advantageous factors like wellbeing and fulfillment and deconstructive relationships to disadvantageous factors like burnout (Wang et al., 2019).

Future research could be done to scrutinize the extent to which other emotions like superiority, nervousness, and boredom are associated with teacher–student relations and emotional exhaustion. Since it is stated that occupational wellbeing is a multifaceted event, and there are individual alterations in educators’ wellbeing, more empirical studies could be carried out to take both teachers’ behavior and individual differences into consideration. Further studies should be done to reveal how the teacher–student relationship is related to alterations in teachers’ occupational wellbeing. The teacher–student relationship displays the main role in teacher wellbeing; therefore, boosting and nurturing such kind of this relationship could maintain their wellbeing which should be assured through conducting more experimental studies in the language learning context. More studies can be done to take other demographic information, such as gender, and teaching experience.

## Author Contributions

The author confirms being the sole contributor of this work and has approved it for publication.

## Funding

This work was supported by Jiangsu Province Academic degree Postgraduate research innovation project, China (grant no:KYZZ16_0104).

## Conflict of Interest

The author declares that the research was conducted in the absence of any commercial or financial relationships that could be construed as a potential conflict of interest.

## Publisher’s Note

All claims expressed in this article are solely those of the authors and do not necessarily represent those of their affiliated organizations, or those of the publisher, the editors and the reviewers. Any product that may be evaluated in this article, or claim that may be made by its manufacturer, is not guaranteed or endorsed by the publisher.
